# CoreProbe: A Novel Algorithm for Estimating Relative Abundance Based on Metagenomic Reads

**DOI:** 10.3390/genes9060313

**Published:** 2018-06-20

**Authors:** Dongmei Ai, Hongfei Pan, Ruocheng Huang, Li C. Xia

**Affiliations:** 1 School of Mathematics and Physics, University of Science and Technology Beijing, Beijing 100083, China; aidongmei@ustb.edu.cn; 2 Sinotech Genomics, Shanghai 200120, China; ruocheng.huang@sinotechgenomics.com; 3 Department of Medicine, Stanford University School of Medicine, 269 Campus Dr., Stanford, CA 94305, USA

**Keywords:** core genome, relative abundance estimation, metagenomics, Gibbs sampling, Dirichlet model

## Abstract

With the rapid development of high-throughput sequencing technology, the analysis of metagenomic sequencing data and the accurate and efficient estimation of relative microbial abundance have become important ways to explore the microbial composition and function of microbes. In addition, the accuracy and efficiency of the relative microbial abundance estimation are closely related to the algorithm and the selection of the reference sequence for sequence alignment. We introduced the microbial core genome as the reference sequence for potential microbes in a metagenomic sample, and we constructed a finite mixture and latent Dirichlet models and used the Gibbs sampling algorithm to estimate the relative abundance of microorganisms. The simulation results showed that our approach can improve the efficiency while maintaining high accuracy and is more suitable for high-throughput metagenomic data. The new approach was implemented in our CoreProbe package which provides a pipeline for an accurate and efficient estimation of the relative abundance of microbes in a community. This tool is available free of charge from the CoreProbe’s website: Access the Docker image with the following instruction: sudo docker pull panhongfei/coreprobe:1.0.

## 1. Introduction

Microbial organisms are ubiquitous in virtually all the natural environments of the earth’s biosphere. They play integral and unique roles in ecosystems [1], they are involved in the biogeochemical cycling of the earth [2], and they have a great impact on human health. There is a dynamic equilibrium between the intestinal microflora, the host, and the environment. Once the structure, composition, and function of the microbiota cause alterations in the metabolites of the intestinal microbiota, there can be host diseases such as obesity [3], malnutrition [4] and diabetes [5]; intestinal flora disorders and irritable bowel syndrome [6], ulcerative colitis and Crohn’s disease [7,8] and other chronic bowel diseases, colon cancer [9,10] and gastric cancer [11]. Thus, profiling the taxonomic composition using the microbial abundances of related communities is critical for understanding the microbial ecology of the environment and for human health.

Recent innovations in metagenomic shotgun sequencing have made it possible to characterize microbial contents in uncultured samples by yielding billions of short reads from metagenomes. Many algorithms have been merged to estimate a community’s taxonomic composition by analyzing the metagenomics sequencing data. These algorithms can be classified into two categories, alignment-based or composition-based, according to their different resolutions of taxonomic binning.

The composition-based algorithms classify metagenomic reads mostly according to their k-mer frequencies. A number of unsupervised methods of this kind have been employed for clustering the reads generated from similar taxonomies. For example, TETRA [12] clusters reads based on Pearson’s correlation coefficients of z-scores, which evaluate the divergence between the observed and expected reads of tetra nucleotide frequencies. CompostBin [13] and SCIMM [14] step up by using a weighted PCA algorithm and an interpolated Markov model, respectively, to bin the reads. MetaCluster [15] involves two phases of clustering to guarantee the accuracy of the read binning. Some supervised methods have also been proposed to consider the available genomic information and to assign taxonomic labels. Various machine learning methods such as the support vector machine (SVM) classifier, naive Bayes classifier, and Gaussian kernel function are employed in Phylopythia [16], NBC [17] and TACOA, respectively [18]. In addition, RAIphy [19] assigns reads taxonomically according to each read’s k-mer index value sum based on its Relative Abundance Index model. Other composition-based methods directly explore the k-mer sequences of the reads, such as LMAT [20], Kraken [21] and CLARK [22], which assigns taxonomic labels using the lowest common ancestor (LCA) approach based on the matches of the reads’ k-mer sequences with their differently self-built k-mer databases. PhymmBL [23] and RITA [24] use hybrid information from BLAST results to improve their accuracy. MetaTopics [25] can efficiently extract the latent microbial communities by using the topic model, and it reflects the intrinsic relations or interactions among several major microbes.

Old composition-based methods such as TETRA and TACOA show sharply reduced accuracy during the abundance estimation when the lengths of the reads are less than 800 bp or the community complexity of the samples is high. Although it is claimed that newly merged composition-based methods can be applied quickly and accurately to metagenomic datasets from next-generation sequencing with read lengths of less than 300 bp, these methods still do not appear to be widely used in real metagenomic analyses because there is currently an open question about how k-mer compositions are associated with microbial phylogeny. More evidence must be discovered until the compositional characteristics of DNA sequences become solid phylogenetic signals.

In the alignment-based approaches, alignment and mapping tools, such as BLAST [26], BWA [27] and Bowtie2 [28], are used to find similarity hits in metagenomic reads to reference sequences and then assign the mapped reads to the most plausible microbial lineages. Early alignment-based methods simply estimate the microbial abundances according to the mapped counts. Most of these methods focus primarily on precisely filtering mapped reads and employing the lowest common ancestor algorithm. Methods such as MEGAN [29], CARMA [30], PaPaRa [31] and MTR [32] all have different strict mapping and filtering procedures or preprocessing procedures to achieve an accurate estimation. Then, the reference length normalization and probabilistic model are introduced into the metagenomic reads analysis. GAAS [33] also improves the microbial abundance estimates by introducing similarity weighting based on alignment e-values. GRAMMy [34] accurately estimates the microbial species abundances based on the mixture model theory and EM algorithm, while Pathoscope [35] presents a complete framework of the metagenomic composition analysis. GASic [36] and MetaMix [37] add additional probabilistic procedures such as similarity correction by Least Absolute Shrinkage and Selection Operator (LASSO) or model comparison by Markov-Chain Monte Carlo (MCMC) to improve the estimation.

Another way to perform microbial abundance estimations other than introducing the probabilistic model to alignment-based approaches is to substitute traditional complete genome sequences with more condensed and informative marker sequences as references. MetaPhlAn [38] and mOTU [39], which employ clade-specific marker genes as reference sequences, have greatly improved the speed and accuracy of analyzing extremely large metagenomic datasets. Clade-specific marker genes can be identified in almost every clade at different microbial taxonomic levels, and thus they are much more representative than 16S rRNA in metagenomic analysis. The use of marker genes as references only accounts for approximately 4% of the sequenced microbial genes, which leads to a significant conservation in alignment time and storage. However, this approach will discard large numbers of reads in the meantime, because most reads will hardly be mapped to marker genes, which is a regretful loss of information from the metagenomic datasets. Moreover, some microbial species may have a rather small percentage of marker genes because of frequent mutations.

Here, we recommend using core genomes as the reference sequences of potential species and estimating the microbial abundances in metagenomic analysis by using the probabilistic model. According to the pan-genome concepts motivated by Tettelin [40], a clade’s core-genome, which contains genes shared by all the strains within the clade, typically includes the genes responsible for the major phenotypic traits, which account for nearly 8% of the genetic repertoire. In involving clade-specific marker genes as a subset, the core-genome generally contains some genes that may be shared by different clades. The reasons for using core-genomes as references are as follows: First, the coexistence of different subpopulations of a microbial species may be a general feature in highly mixed habitats. Evidence has been discovered of both selected cultured isolates and wild uncultured populations, from marine environments [41] to human body sites [42]. The existence of multiple subpopulations or strains, the abundances of which are very likely to vary greatly, often brings extensive genomic diversity and community complexity and leads to inaccurate results when using only several complete genomes or a small percentage of marker genes as references for a clade. However, using core-genomes that consist of the genes shared by all the microbial strains that were studied as references provides us with an opportunity to estimate the relative abundance of a species with a complex substrain composition quickly and accurately. Second, variational subpopulations of a microbial species that have never been sequenced before, the strains of one species that may have high similarity with another species [43], and some mechanisms such as horizontal gene transfer and lysogeny, which currently tend to be considered to occur more frequently in natural environments than previously thought [44], are all obstacles to alignment accuracy when using the strains’ complete reference genomes. Otherwise, using core-genomes as references should include the general new strains of the species because most currently identified core genes should also be shared by those strains. Different microbial species with high similarity or exotic sequences would also be addressed because they would differ significantly in their core-genomes. Third, compared to using marker genes as clade references, the shared or redundant genes between different clades in the core-genomes form the basis for introducing probabilistic models, which create a balance between mapping quickly and precisely to the references and utilizing the information hidden in ambiguous reads.

In this paper, we introduce CoreProbe, a relative abundance estimation framework for microbes that employs microbial core-genomes as references for metagenomic analysis. In addition, CoreProbe takes advantage of the mixture model theory and describes the sequencing procedure for metagenomic reads as a generative model to accurately estimate microbial abundances with the Gibbs sampling algorithm [45,46,47]. We first tested CoreProbe both in our own simulated metagenomic read sets using MetaSim [48] and in third-party synthetic communities [49]. From these experiments, we can observe that using core-genomes as references in CoreProbe sharply outperforms the use of ordinary complete microbial genomes, whether the specific strains of the reference genomes are “in” the metagenomic datasets or not. Compared to other methods including GRAMMy [34], Pathoscope [35], MetaPhlAn [38] and Kraken [21], CoreProbe also shows improved accuracy in abundance estimations. We then analyzed 25 real metagenomic read sets from Human Microbiome Project (HMP) (https://portal.hmpdacc.org/), yielding new insights into microbiomes from different human body sites. Finally, we implemented CoreProbe in C++, and we accessed the Docker image with the following instruction: sudo docker pull panhongfei/coreprobe:1.0.

## 2. Methods

### 2.1. A Finite Mixture and Latent Dirichlet Model

To estimate the relative abundance of reference species accurately according to a metagenomic dataset, we describe the sampling and sequencing procedure of metagenomic reads as a generative model: First, in using M microbial organisms as the reference species, the metagenome $\bar{M}$ in a metagenomic dataset can be denoted as

(1) $$\bar{M}=\sum_{i=1}^{M}\theta_{i}g_{i}$$

where $\{g_{1},g_{2},\cdots ,g_{M}\}≜G$ represents the reference sequences of M known species. These reference sequences can be contigs, complete genomes, pan-genomes, core-genomes, etc. $(\theta_{1},\theta_{2},\cdots ,\theta_{M})≜\vec{\theta }$ denotes the mixture parameters of those reference sequences (or reference species). It should be noted that

(2) $$\sum_{i=1}^{M}\theta_{i}=1$$

and each $\theta_{i}$ is proportional to its relative species abundance $a_{i}$ and the corresponding reference sequence’s base length $l_{i}$, i.e., $\theta_{i}\propto a_{i}l_{i}$ according to Xia et al. [33]. Here, those organisms are subject to a particular discrete distribution $\left(\theta_{1},\theta_{2},\cdots ,\theta_{M}\right)$ in the metagenomic generative model. Second, from metagenome M, we randomly chose reference species $g_{i}$, which is subject to the multinomial probability $\theta_{i}$, because each read must be generated from the biological sequence of a particular species. Third, given the chosen genome $g_{i}$, we randomly generated read $r_{k}$. The generation of reads from the reference species $g_{i}$ is subject to $g_{i}$’s read-composition distribution

(3) $$(\varphi_{r_{1},g_{i}},\varphi_{r_{2},g_{i}},\cdots ,\varphi_{r_{K},g_{i}})≜{\vec{\varphi }}_{i}$$

where $\varphi_{r_{k},g_{i}}$ denotes the probability of generating a particular read $r_{k}$ from genome $g_{i}$, i.e., $p\{read\_k|G=g_{i}\}$. Here, we assume K, the total number of different reads generated from metagenome $\bar{M}$. We denote the set of the read-composition distributions for all the reference species as $\{{\vec{\varphi }}_{g_{1}},{\vec{\varphi }}_{g_{2}},\cdots ,{\vec{\varphi }}_{g_{M}}\}≜\Phi$.

Based on the assumptions in the metagenomic generative model, we can easily induce the formula of the relative abundances for reference species when their reference sequence lengths $\left\{l_{1},l_{2},\cdots ,l_{M}\right\}$ and mixture parameters $\left(\theta_{1},\theta_{2},\cdots ,\theta_{M}\right)$ are known. Noting that $\sum_{i=1}^{M}\theta_{i}=1$ and $\theta_{i}\propto a_{i}l_{i}$, the relative abundance formula of the reference species under the metagenomic generative model is as follows:

(4) ai=θili∑s=1Mθsls

The procedure in the metagenomic generative model will be repeated N times to obtain N metagenomic reads. We can then use the obtained reads to infer the mixture parameters and then the relative abundances of the reference species based on the generative model. As we can observe, both sampling procedures for the second and third steps are subject to multinomial distributions, i.e., $g_{i}∼Mult(\vec{\theta })$ and $r_{k}∼Mult({\vec{\varphi }}_{g_{i}})$, respectively. For the sake of the calculation, we followed the suggestion of Pritchard [45] and used the Dirichlet distribution as the prior distribution of species mixture parameters in the metagenome because of the Dirichlet-multinomial conjugacy. However, we do not assume prior distributions for the read-composition distributions Φ of those reference species, because we can approximate their full conditional probabilities during parameter inference via read-to-reference-sequence alignment results. The above generative model for metagenomic reads is shown as the pseudo-code in Algorithm 1.

**Algorithm 1** (A finite mixture and latent Dirichlet model for metagenomics).
***Require** the hyperparameter*
$\vec{\alpha }$
*, the total number of reads*
N
*, the species set*
G
*, the read-composition distributions*
Φ

***Ensure***

*the read dataset R*

*sample the species mixture parameters*
$\vec{\theta }∼Dir(\vec{\alpha })$
*for a metagenome*

***repeat***

*1. sample species*
$g_{i}∼Mult(\vec{\theta })$

*2. sample read*
$r_{k}∼Mult({\vec{\varphi }}_{g_{i}})$

***until***
*the total number of metagenomic reads*
N
*is reached*

***return***
*the read dataset*
$\text{\{}r_{1},r_{2},\cdots ,r_{N}\text{\}}≜R$


### 2.2. Mixture Parameter Inference and Gibbs Sampling

Ideally, we would need exact knowledge about the occurrence counts of the read-origin species for each read in a metagenomic dataset to estimate the mixture parameters $\vec{\theta }$ accurately. However, this task is basically impossible to perform through sequence alignment due to the inherent ambiguity of relatively short NGS reads and the complex microbial communities, which generally consist of species with similar reference genomes. Hence, we employ a parameter inference procedure involving Gibbs sampling to infer the mixture parameter.

In this section, we introduce hidden variables $(z_{1},z_{2},\ldots z_{N})≜\vec{z}$ for the first time, where $z_{i}$ is an index denoting the reference species that generates the corresponding read $r_{i}$. We develop an approximate inference algorithm of Gibbs sampling to emulate the probability distribution of $z_{i}$ given the observations of metagenomic reads R conditioned on prior probability $\vec{\alpha }$ and the read-composition distributions Φ of those reference species; i.e., $p\{\vec{z}|R;\vec{\alpha },\Phi \}$. We can then statistically estimate the counts of the reference species using the samples of $p\{\vec{z}|R;\vec{\alpha },\Phi \}$ after the burn-in period of Gibbs sampling, and herewith obtain the estimation of parameters $\vec{\theta }$ and then the relative species abundances $\vec{\alpha }$. Specifically, Gibbs sampling generates an instance of each dimension $z_{i}$ of $\vec{z}$ in turn, subject to their full conditional $p\{\vec{z}|{\vec{z}}_{\neg i},R;\vec{\alpha },\Phi \}$, where ${\vec{z}}_{\neg i}$ denotes all other dimensions of $\vec{z}$ except $z_{i}$. It can be shown [50] that under this condition, the sequence of samples $\left\{{\vec{z}}_{1},{\vec{z}}_{2},\ldots \right\}$ constitutes a Markov chain whose stationary distribution is $p\{\vec{z}|R;\vec{\alpha },\Phi \}$. During the real application of metagenomic data, we can extract a certain number of samples of $\vec{z}$ after the burn-in period, and we can calculate the average counts of reference species to infer the parameters.

Next, we derive the full conditional $p\{\vec{z}|R;\vec{\alpha },\Phi \}$ for Gibbs sampling. First, we calculate the probability $p\{\vec{z}|\vec{\alpha }\}$. Starting with the probability $\vec{z}$ as conditioned to the species mixture probability $\vec{\theta }$, and noting that the hidden species indices are generated as multinomial trials, we have

(5) $$p\{\vec{z}|\vec{\theta }\}=\prod_{i=1}^{M}\theta_{i}^{n_{i}}$$

where $n_{i}$ refers to the number of reads whose corresponding species index is i. Noting that we assume $p\{\vec{\theta }|\vec{\alpha }\}$ is subject to Dirichlet distribution, we have

(6) $$\int p\{\vec{\theta }|\vec{\alpha }\}d\vec{\theta }=\int \frac{1}{△(\vec{\alpha })}\prod_{i=1}^{M}\theta_{i}^{\alpha_{i}-1}d\vec{\theta }=1$$

where

(7) $$△(\vec{\alpha })=\frac{\prod_{k=1}^{M}\Gamma (\alpha_{k})}{\Gamma (\sum_{k=1}^{M}\alpha_{k})}$$

Then, by using the above two formulas, and integrating out $\vec{\theta }$ in the following conditional probability formula, we obtain

(8) $$p\{\vec{z}|\vec{\alpha }\}=\int p\{\vec{z}|\vec{\theta }\}p\{\vec{\theta }|\vec{\alpha }\}d\vec{\theta }=\int \frac{1}{△(\vec{\alpha })}\prod_{i=1}^{M}\theta_{i}^{n_{i}+\alpha_{i}-1}d\vec{\theta }=\frac{△(\vec{n}+\vec{\alpha })}{△(\vec{\alpha })}$$

Second, we obtain the probability of reads conditioned on the hidden variables $\vec{z}$ with the knowledge of read-composition distribution set Φ as

(9) $$p\{\vec{r}|\vec{z};\Phi \}=\prod_{i=1}^{N}p\{r_{i}|z_{i},\Phi \}=\prod_{i=1}^{N}\phi_{r_{i},g_{i}}=\prod_{k=1}^{K}\prod_{m=1}^{M}{\left(\phi_{r_{k},g_{m}}\right)}^{n_{\left[k,m\right]}}$$

where we assume that the generation of each read is independent of other reads, K is the number of different reads, M is the number of reference species, and $n_{\left[k,m\right]}$ is the count of reads that have the same sequence as $r_{k}$ and whose corresponding species is $g_{m}$. In real applications, we can estimate $\phi_{r_{i},g_{i}}$ by finding the ratio of high-quality hits for $r_{i}$ to all the high-quality read hits on the target reference sequence $g_{i}$ from the alignment result; that is,

(10) $$\phi_{r_{i},g_{i}}\approx \frac{\#\;of\;r_{i}\;that\;hit\;g_{i}\;with\;high\;quality}{\#\;of\;reads\;that\;hit\;g_{i}\;with\;high\;quality}$$

Thus, we have

(11) $$\begin{array}{ll} p\left\{z_{i}=t|{\vec{z}}_{\neg i},R;\vec{\alpha },\Phi \right\} & =\frac{p\left\{\vec{z},\vec{r}\right\}}{p\left\{{\vec{z}}_{\neg i},\vec{r}\right\}}\\ & =\frac{p\left\{\vec{r}|\vec{z}\right\}p\left\{\vec{z}\right\}}{p\left\{{\vec{r}}_{\neg i}|{\vec{z}}_{\neg i}\right\}p\left\{{\vec{z}}_{\neg i}\right\}p\left\{r_{i}\right\}}\\ & \propto \frac{p\left\{\vec{r}|\vec{z}\right\}}{p\left\{{\vec{r}}_{\neg i}|{\vec{z}}_{\neg i}\right\}}\frac{p\left\{\vec{z}\right\}}{p\left\{{\vec{z}}_{\neg i}\right\}}\\ & =\frac{\prod_{k=1}^{K}\prod_{m=1}^{M}{\left(\phi_{r_{k},g_{m}}\right)}^{n_{\left[k,m\right]}}}{\prod_{k=1}^{K}\prod_{m=1}^{M}{\left(\phi_{r_{k},g_{m}}\right)}^{n_{[k,m],\neg i}}}\frac{△(\vec{\alpha }+\vec{n})}{△(\vec{\alpha }+{\vec{n}}_{\neg i})}\\ & =\phi_{r_{i},g_{t}}\cdot \frac{\left(n_{t,}{}_{\neg i}+\alpha_{i}\right)}{\sum_{j=1}^{M}\left(n_{j,}{}_{\neg i}+\alpha_{j}\right)}\\ & \propto \phi_{r_{i},g_{t}}\cdot \left(n_{t,}{}_{\neg i}+\alpha_{i}\right) \end{array}$$

Here, we leave the priors out to simplify the notations. The relation Γ(a+1)=aΓ(a) is used in the above formula, and ¬i indicates that the number is counted exclusive of the read $r_{i}$. $n_{\left[k,m\right]}$ is the count of reads that have the same sequence as $r_{k}$ and whose corresponding species is $g_{m}$. $n_{\left[k,m\right]}$ and $n_{[k,m],\neg i}$ are different only when k=i,m=t in the above formula, and $n_{\left[i,t\right]}=n_{[i,t],\neg i}+1$. $\vec{n}$ indicates the occurrence counts of read-origin species in the metagenomic reads, whose element

(12) $$n_{j}=n_{\left[\cdot ,j\right]}=\sum_{s=1}^{M}n_{\left[s,j\right]}$$

Similarly, $\vec{n}$ and ${\vec{n}}_{\neg i}$ are the same except for their tth element in the above formula, and $n_{t}=n_{t,\neg i}+1$. The above formula shows that read $r_{i}$ is more likely to be assigned to reference species $g_{t}$ if the probability that $g_{t}$ generates $r_{i}$ is large and if there are many reads in the metagenomic datasets that have been assigned to $g_{t}$.

Finally, we can infer the genome mixture parameters $\vec{\theta }$ using the counts vector $\vec{n}$. For the Dirichlet-multinomial conjugacy, we have

(13) $$p\{\vec{\theta }|\vec{z},\vec{\alpha }\}=Dir(\theta |\vec{n}+\vec{\alpha })$$

and we can estimate $\vec{\theta }$ from the expectation of its distribution as follows:

(14) $$\theta_{k}=\frac{n_{k}+\alpha_{k}}{\sum_{i=1}^{M}\left(n_{i}+\alpha_{i}\right)}$$

$\vec{n}$ can be statistically estimated by finding the mean of a certain number of samples after the burn-in period of Gibbs sampling described above to overcome the ambiguity of the reads. The abundances can then be estimated. The pseudo-code of Gibbs sampling is shown in Algorithm 2.

**Algorithm 2** (Gibbs Sampling Algorithm for the Metagenomic Model).
***Require:***
*references species*
M
*, metagenomic reads*
R
*, hyperparameter*
$\vec{\alpha }$

***Global data:***
*count statistics*
$\vec{n}$
*, read-composition distributions*
Φ
*, memory for full conditionals*
$p\{z_{i}|{\vec{z}}_{\neg i},R;\vec{\alpha },\Phi \}$

***Ensure:***
*mixture parameters*

$\vec{\theta }$
*//initialization: obtain read-composition distributions*
Φ
*according to alignment results zero all count statistics*
$\vec{n}$

***for***
i=1
*to*
N
***do** sample the species index*
$z_{i}=m∼Mult(M)$
*increment sampled species count*
$n_{m}=n_{m}+1$

***end for***
*//Gibbs sampling*

***while***
*not finished **do***

***for***
i=1
*to*
N
***do***
*decrement target species count*
$n_{m}=n_{m}-1$

*sample a new species index*
$z_{i}=\tilde{m}∼p\{z_{i}|{\vec{z}}_{\neg i},R;\vec{\alpha },\Phi \}$

*increment sampled species’ count*
$n_{\tilde{m}}=n_{\tilde{m}}+1$

***end for***

***if***
*converged and a given number of samples generated **then***

***return***
*mixture parameter*
$\vec{\theta }$
*according to the equation*

***end if***

***end while***


## 3. Results

### 3.1. The CoreProbe Framework

**Figure 1:**
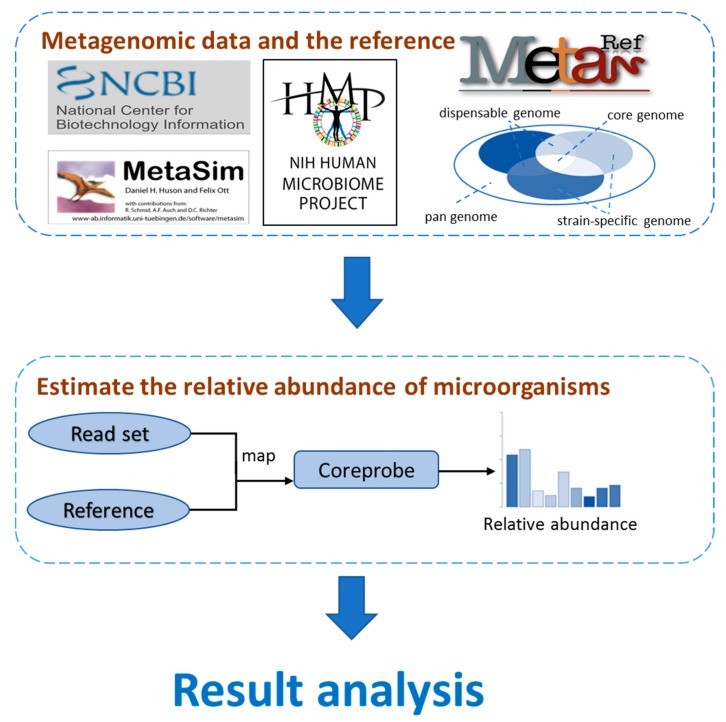
Overview of the overall content of the article. We first downloaded the reference sequence from the National Center for Biotechnology Information (NCBI) database (https://www.ncbi.nlm.nih.gov/), the MetaRef database, and simulated the metagenomic data with MetaSim software [48]. We then used our CoreProbe to analyze the simulated data to estimate the genome relative abundance, to compare them with the existing methods to assess the accuracy and efficiency of our method. Last, our method was applied to the actual data obtained from Human Microbiome Project (HMP), and a preliminary analysis was made to support the practicability of our method.

The primary contents of this article can be summarized as shown in Figure 1.

**Figure 2:**
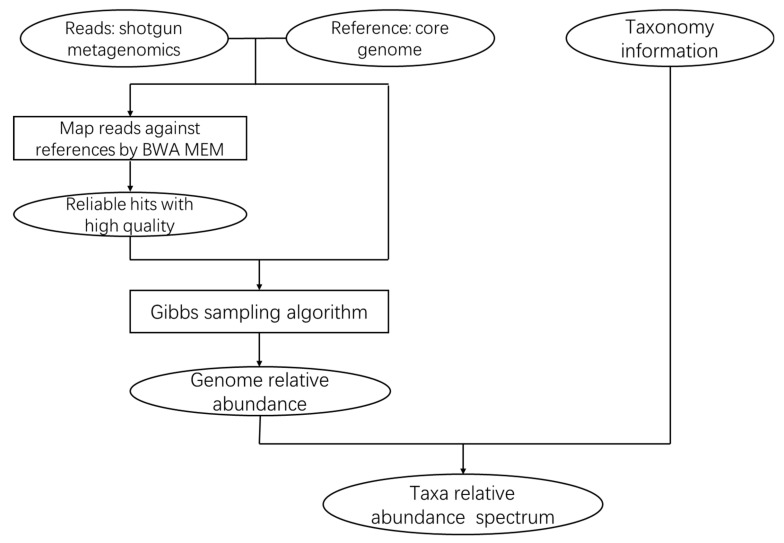
The CoreProbe framework. First, the metagenomic reads obtained by shotgun sequencing method was compared with the core genome reference sequence using BWA [27], and the results of the alignment were recorded. The results were then combined with our reads set and the reference sequences applied to the Gibbs sampling algorithm to obtain the relative abundance of the metagenome. Finally, if the taxonomic information for the input reference genomes was available, we could calculate a high level of taxonomic abundance combined with the strain level estimates.

We then created a brief introduction to the specific CoreProbe process, which can be found in Figure 2.

### 3.2. Simulation Result

To evaluate the performance of the CoreProbe framework and compare it with the existing methods, we generated 90 simulated metagenomic datasets. We chose 40 microbial species as embed microorganisms in simulated metagenomic datasets. We aimed to estimate the relative abundances of 10 species, leaving the others as “unknown” species, and we compared the precision of the estimations among the existing methods. We downloaded the current completely sequenced microbial genomes of those species in which sequences may coexist, representing different strains of one species. We then employed MetaSim [48] to generate simulated reads of the selected genomes with preset relative abundances. Specifically, we built an Empirical Error Model based on the study of the Illumina’s sequencing technology [51,52,53] to generate metagenomic datasets that contained 1000, 2000, 5000, 10,000, 20,000, 50,000, 100,000, 200,000, and 500,000 single-ended reads with an average length of approximately 100 bp, and each dataset had 10 replicates.

When applying CoreProbe to those simulated datasets, we first downloaded all the comprehensive non-redundant reference core gene catalogs, which contained classification information and their corresponding sequences, and they also included over 2800 microbial species. Second, we selected certain microbial species as reference species, and we extracted all the core gene sequences for each reference species in the downloaded core gene catalogue. Third, we concatenated the core gene sequences of a reference species together into one FASTA file as a reference sequence of the species. We then used BWA MEM to map the simulated metagenomic reads against the reference core-genome sequences. All parameters were set as the defaults, and the output consisted of all the alignments in a SAM file.

The output SAM files were then passed to our program to estimate the relative abundances. In addition, we applied the three widely used algorithms for an abundance estimation, including GRAMMy [34], MetaPhlAn [38] and Kraken [21], and then used the microbial whole genome sequences as references for the metagenomic analysis to process the above 90 groups of metagenome simulation data and to estimate the relative abundance of 10 species of microorganisms. In addition, we compared the results using the CoreProbe algorithm to compare the accuracy and speed, to support the effectiveness of this algorithm.

It should be noted that when estimating the relative abundance of microorganisms, two types of whole genome sequenced strains were selected as the reference genome of the above algorithm, namely, (1) the selected strain used as a reference genome is contained in a sequence of strains used to generate simulated metagenomic datasets as $g_{1}$; and (2) the selected strain used as a reference genome is not included in the strain that is used to generate the simulated metagenomic datasets, and it is recorded as $g_{2}$. This classification is because there are often new strains of certain microbial species in the natural environment, and the sequence has not been effectively sequenced. The use of as a reference genome is used to simulate these cases.

#### 3.2.1. Comparison of Algorithm Accuracy

**Figure 3:**
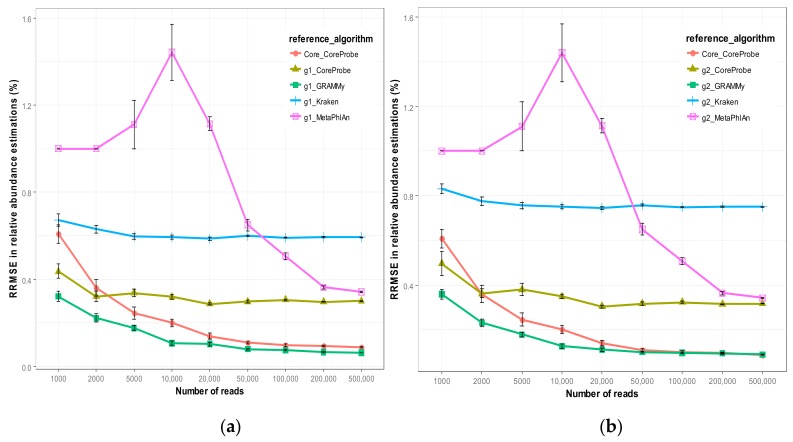
The relative mean square error of the relative abundance of each algorithm. (**a**) The selected strain as a reference genome is contained in a sequence of strains used to generate simulated metagenomic datasets as $g_{1}$; and (**b**) the selected strain as a reference genome is contained in a sequence of strains used to generate simulated metagenomic datasets as $g_{2}$. The abscissa represents the different metagenome simulation data set of the reading capacity, and the ordinate is the mean square error of the algorithm relative abundance between the estimated value and the preset value.

We compare the accuracy by using the relative mean square error of the relative abundance of each algorithm, and the results are shown in Figure 3a,b.

There are some common characteristics of the two graphs. With the increased capacity of the metagenome reads (i.e., the improvement of the depth of the sequencing), the mean variance of the relative abundance estimation of each algorithm decreases, indicating that the increase in the sequencing depth can improve the accuracy of each algorithm. MetaPhlAn [38] is rather special, in that the mean variance in the relative abundance values obtained here will begin to rise and then quickly decrease with the increase in the sequencing depth. When the metagenome reading capacity is less than 10,000, the relative abundance of the estimated variance is even more than 100%, indicating that with the increased depth of the sequencing, the accuracy will decrease; and when the reading capacity continues to increase, its mean square error will decline rapidly. Especially when the capacity of reads reaches up to 50,000, MetaPhlAn [38] is more accurate than Kraken [21]. Thus, we can observe that the MetaPhlAn algorithm [38] is not stable from the point of view of the sequencing depth, though it achieves high accuracy when the sequencing depth is sufficient. Kraken [21] is rarely affected by sequencing, with a mean square deviation remaining between 60% and 70%, indicating that the accuracy of the Kraken [21] is not high; there is a certain gap between the calculation results and the actual value. GRAMMy [34] has the highest accuracy; its variance is below 40%, and decreases occur with the increasing sequencing depth. The CoreProbe algorithm mentioned in this paper also uses the whole genome for the reference sequence. Its accuracy is better than that of MetaPhlAn [38] and Kraken, but there is still a gap with the GRAMMy algorithm [34]. However, when CoreProbe uses the core-genome sequence as the reference sequence, the accuracy is significantly improved. When the metagenome reads capacity is increased to 50,000, the accuracy is very close to that of GRAMMy [34], and the mean square error in the preset value is less than 10%.

However, by comparing Figure 3a and Figure 3b, the accuracy of each algorithm was found to decrease when the reference genome changes from $g_{1}$ to $g_{2}$. Although the difference is not large, the selected reference genome sequence corresponds to the strains present in the sample environment or not, and they will have some impact on the algorithm. In this paper, CoreProbe uses the core gene sequence as the reference sequence, and thus, the variations in the strain in the real environment and the unknown strain that affect the algorithm are relatively small. Therefore, the relative abundance estimation is made using the microbial core genome as the reference. When the actual environment has not yet sequenced new strains, the algorithm estimates of the relative abundance for the accuracy can be more credible.

#### 3.2.2. Comparison of Algorithm Speeds

**Figure 4:**
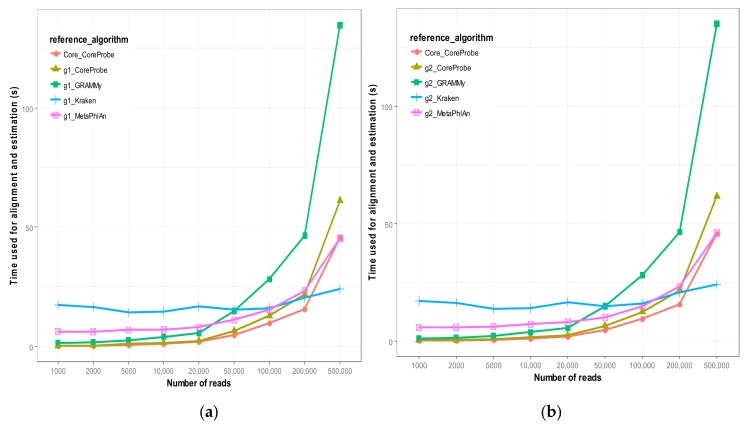
The time used for each algorithm. (**a**) The time used for each algorithm in different reads capacities with $g_{1}$ as the reference genome. (**b**) The time used for each algorithm in different reads capacities with $g_{2}$ as the reference genome. The abscissa represents the different metagenome simulation datasets of the reads capacity, and the vertical coordinate is the time spent on the calculation process of each method.

The various algorithms mentioned in this paper are used in different algorithms, and during the process of dealing with data, there are different sequence mapping algorithms; for example, Kraken [21], MetaPhlAn [38], and GRAMMy [34] correspond to BLAST [26], Bowtie2 [28], and BWA [27]. Thus, the time spent on the calculation process (the sum of time used by the sequence alignment and the statistical algorithm) will be different, and we used the line graph to show the efficiency of each algorithm, as shown in Figure 4a,b.

The overall situation of the two figures is similar. As shown in Figure 4a,b, with the increased capacity of the metagenome (i.e., the improvement of the depth of the sequencing), the relative abundance estimation time of each algorithm is increased. However, the Kraken [21] is special, because the time it consumes does not increase as the depth of the sequencing increases, and the time was almost maintained at approximately ten seconds. GRAMMy [34] is the most accurate, but it is time-consuming, and especially when the sequencing depth increases, the growth is faster than it is in other algorithms. MetaPhlAn [38] is significantly more time-consuming than the other algorithms, and the computational accuracy is lower than that of other algorithms. When the sequencing depth increases, its required time starts to increase significantly, and its accuracy also starts to improve significantly. In addition, our method, CoreProbe, is the least time-consuming, and it only needs more time than Kraken [21] when the number of reads goes up to 200,000. At the same time, by comparing Figure 4a and Figure 4b when the reference genome changes from to , the speed of each algorithm is almost unaffected. This finding shows that each algorithm is relatively stable, and when the amount of data is equivalent, the calculation process will not change the necessary time. Because the microbial core genome is the gene that is common to all the strains in the clade, the number is much smaller than that of the whole genome, and thus the computational time can be dramatically reduced and the relative abundance can be estimated more efficiently. As shown in the figure, when the core genome was used as a reference sequence, the time consumption was less than that of the metagenomes $g_{1}$ and $g_{2}$.

### 3.3. Real Metagenomic Datasets Analysis

As an immediate application, we applied this CoreProbe pipeline to a set of actual data. These data come from the HMP, and its sub-database HMIWGS contains 764 groups of high-throughput metagenomic sample data from 16 different human tissues via the Illumina platforms. In this paper, five different human tissues were selected, including the anterior nares, buccal mucosa, posterior fornix, right retro-auricular crease, stool, and five different genomic samples that were randomly selected from each organization. The metagenome data in this paper have passed the quality control (QC) test of the HMP. We intended to use these data to estimate the relative abundance of microorganisms and to classify the metagenomic data. Thus, no additional preprocessing of the above metagenome data was performed.

In this section, 150 common microbes were selected, and the gene sequences belonging to their core genomes were selected from the MetaRef database. Their core gene sequences were grouped together to form a FASTA formatted file as the reference genome sequence of the corresponding microorganism. In this paper, we used the BWA MEM program to compare the sequence of the actual genome reading with the reference genome. This parameter was used to locate the default value and output all the matching results. Finally, the CoreProbe algorithm implemented in this paper was used to estimate the relative abundance of microbes in each sample.

**Figure 5:**
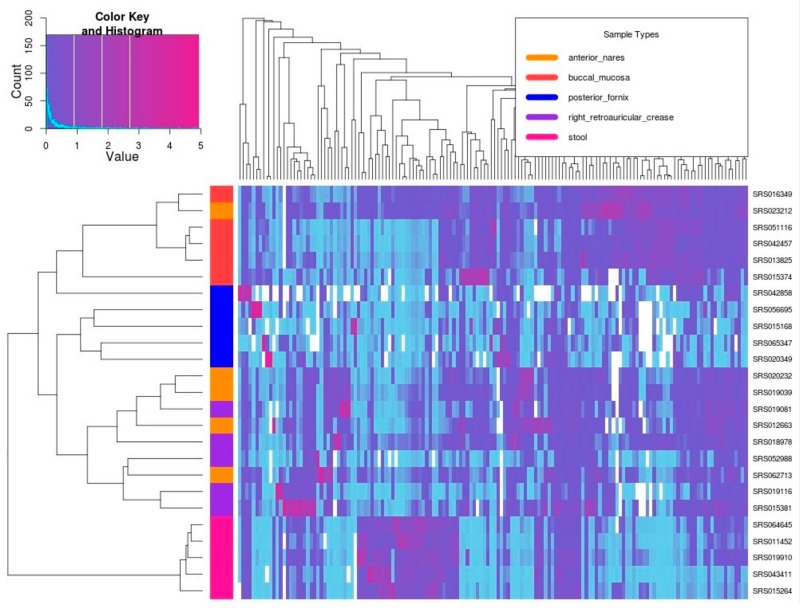
Thermal graph of microbial species relative abundance data in five different human tissues. The horizontal axis corresponds to the different microbial species, and the vertical axis corresponds to the metagenome samples of different human tissues. The white cells indicate that the microorganism corresponding to its vertical axis does not appear in the corresponding sample on the horizontal axis. The change in color from light blue to deep purple indicates that the relative abundance of microorganisms in the corresponding sample varies from low to high. The distance used by the cluster analysis of different metagenomic samples on the left side of the thermogram is derived from the Spearman correlation coefficient between the relative microbial abundance vectors (using the difference between unit 1 and the correlation coefficient as a distance).

On this basis, the relative abundance of the obtained microbes was analyzed by heat map. In Figure 5, the buccal mucosa, vaginal dome and stool samples were basically clustered into one class, indicating that the relative abundance of microbes showed similarities within their respective tissues and had significant differences from other human tissues, which is similar to the results of other studies [54]. In addition, the anterior nares and the right retro-auricular crease corresponding to the metagenomic samples have been doped, and they cannot be clustered well, indicating that the relative microbial abundance between the two human tissues is of great similarity, which may result from close contact between the anterior nares and the right retro-auricular crease and the lesser secretion of body fluid. In addition, there are more white cells relative to the posterior fornix, indicating that the microbial species of this tissue is less than that of others; a small number of cells presented a violet color, and the rest were generally shallow, indicating that the tissue often consists of a small number of microorganisms as the dominant population, and the remaining microbial content is scant. Studies have shown that this finding is due to the important position of the female vagina in the human reproductive system, and its need to inhibit the growth of microbial species. In addition, according to its dominant microbial species, they can be divided into three types of microbial community types [55]. In the classification tree on the left side of the thermogram, it is also clear that the posterior fornix sample is divided into three sub-trees.

Through the above analysis, we can see that the CoreProbe algorithm can accurately and quickly estimate the relative abundance of microbes and analyze them by heat map, which can distinguish among the different genomic samples from different tissues, and it can be used to find the differences from and links in microbial distributions between different tissues. It is of great significance to understand the relationship between human health and microorganisms.

## 4. Discussion

We developed the CoreProbe framework to estimate the relative abundance of genomes. This approach has three unique advantages. First, a complete probability model is established for the relative abundance of microbes. The distribution and structure of microbes are simulated on the basis of this finding; thus, the relative abundance of various microorganisms can be estimated more accurately.

Second, CoreProbe uses the stochastic algorithm of the Gibbs sampling algorithm as an alternative to the deterministic algorithm of statistical reasoning (such as the expectation maximization algorithm) to achieve a better fuzzy allocation of the readings, which makes the method particularly suitable for short read data sets. The distribution of ambiguous sources may be sequencing errors, genetic variation, horizontal gene transfer or closely related genomes. The Gibbs sampling algorithm is a good solution to this series of problems.

Third, due to the changes in microorganisms in the actual environment to adapt to the changes in the surrounding ecology, an abnormal mutation mechanism is evolved, and the genomic polymorphism at the strain level is not uncommon. An environment often contains different strains of a certain microorganism, and the relative abundance of these strains is inconsistent. For the strains that have never been found and sequenced, these factors have greatly affected the accuracy of the relative abundance statistics algorithm for the genome sequence of the few sequenced strains as the reference genome. The core genome is a collection of all the genes of all the strains, which maintain high stability during microbial variation [56].

In using the microbial core genome as a reference genome, the effect of genomic polymorphism and strain diversity on the sequence alignment process can be eliminated to a greater extent, and the accuracy of the relative abundance estimation can be improved. In addition, the core genome can be used as a common gene set of all the strains, accounting for only approximately 8% of all the gene sets of the microbial species. Compared with the algorithm that uses the whole genome of the sequenced strain as a reference gene, the efficiency of the comparison is greatly improved.

In summary, the CoreProbe method we provide is likely to provide a more accurate and efficient estimate of the relative abundance estimates of microbes and to uncover a new direction for estimating abundance.
